# 

**Published:** 1910-07

**Authors:** 


					﻿Vol. XXVI.
JULY, 1010.
No. 1.
TEX J
MEDICAL,
JOURNAL
“Red Back”
PUBLISHED AT AUSTIN, TEXAS. BY
F. E. DANIEL,. M. D„ - - - - Proprietor.
PRINTED BY THE VON BOECKMANN-JONES CO.
Subscription, SI .00 a Year, in Advance. -	-	-	» Single Copies, 10 Cents
Entered at the Postoffice at Austin, Texas, as second class mail matter.
				

